# Involvement of the atrial natriuretic peptide in cardiovascular pathophysiology and its relationship with exercise

**DOI:** 10.1186/1755-7682-5-4

**Published:** 2012-02-07

**Authors:** Júlio C de Almeida, Clodoaldo L Alves, Luiz Carlos de Abreu, Monica A Sato, Fernando L Fonseca, Carlos B de Mello Monteiro, Luiz Carlos M Vanderlei, Hugo Macedo, Carlos M Tavares, Dafne Herrero, Luciano MR Rodrigues, Vitor E Valenti

**Affiliations:** 1 Departamento de Morfologia e Fisiologia, Faculdade de Medicina do ABC. Av. Príncipe de Gales, 821. 09060-650, Santo André, SP, Brazil; 2 Escola de Artes, Ciência e Humanidades da Universidade de São Paulo (USP). Rua Arlindo Béttio, 1000, São Paulo, SP. 03828-000, Brazil; 3 Departamento de Fisioterapia, Faculdade de Ciências e Tecnologia, Universidade Estadual Paulista, UNESP. Rua Roberto Simonsen, 305. 19060-900, Presidente Prudente, São Paulo, Brazil; 4 Departamento de Saúde Coletiva, Universidade Federal do Acre. Campus Universitário, BR 364, Km 04. 69915-900, Rio Branco, AC, Brasil; 5 Departamento de Fonoaudiologia, Faculdade de Filosofia e Ciências, Universidade Estadual Paulista, UNESP. Av. Higyno Muzzi Filho, 737. 17.525-900, Marília, SP, Brazil

## Abstract

In this minireview we describe the involvement of the atrial natriuretic peptide (ANP) in cardiovascular pathophysiology and exercise. The ANP has a broad homeostatic role and exerts complex effects on the cardio-circulatory hemodynamics, it is produced by the left atrium and has a key role in regulating sodium and water balance in mammals and humans. The dominant stimulus for its release is atrial wall tension, commonly caused by exercise. The ANP is involved in the process of lipolysis through a cGMP signaling pathway and, as a consequence, reducing blood pressure by decreasing the sensitivity of vascular smooth muscle to the action of vasoconstrictors and regulate fluid balance. The increase of this hormone is associated with better survival in patients with chronic heart failure (CHF). This minireview provides new evidence based on recent studies related to the beneficial effects of exercise in patients with cardiovascular disease, focusing on the ANP.

## Background

The atrial natriuretic peptide (ANP) has a broad homeostatic role and exerts complex effects on the cardio-circulatory hemodynamics. It influences renal function and lipid metabolism. Its discovery provided a powerful blood propulsion pump to the heart, besides its important endocrine functions. The ANP is produced from a prohormone (pro-ANP), a molecule of 126 amino acids secreted primarily by atrial myocytes after increased tension of the atrial wall [1].

The ANP is produced by the left atrium and has a key role in regulating sodium and water balance in mammals and humans [1,2]. The dominant stimulus for its release is atrial wall tension, a frequent phenomenon occurring during the changes that occur within the cardiovascular exercise. In order to restore and maintain cardiovascular homeostasis the heart synthesizes peptides and hormones with diuretic, natriuretic and vasodilator properties [3,4].

It was observed that concentrations of circulating ANP increase during mild exercise, and during the growing intensity of the maximum levels the concentration reach values up to two to three times higher. These increased concentrations may be related to cardiac functional impairment in people with chronic heart failure (CHF) who are submitted to exercise, in which it was observed that the concentration of this hormone was more evident compared to healthy people [5]. Exercise is a non-pharmacologic way to treat many cardiovascular diseases [6-9], hence, further studies investigating non-pharmacological therapies such as exercise, are always welcome in the literature.

In view of the above considerations, in this minireview we described the involvement of the ANP in cardiovascular pathophysiology and its relationship with exercise.

## Method

The Medline (via PubMed), Lilacs and Scielo databases were searched using the following subject keywords: "Atrial natriuretic peptide," "cardiovascular" and "pathophysiology". We also used the "related articles" function on PubMed, Lilacs and Scielo, which allowed us to search the references of the studies that were retrieved during our search. Publications were included in our review if either their titles or abstracts were available in English or Portuguese and suggested any effect (i.e., beneficial or malefic) or lack thereof from an anti-hypertensive treatment on cardiac hypertrophy. The review was completed on June, 2011. Publications were excluded if the treatment was limited to a particular technique or if the population received only one specific procedure (drug treatment) associated with a disease state or an age group. Other studies on anti-hypertensive treatment for hypertensive patients that offered additional relevant information found in the same database were also examined. Each publication was reviewed to identify the author(s), study period and data source, which influenced us to include the reference in the study.

## Results and discussion

Table 1 shows the main studies related to pathophysiological mechanisms of the ANP and its involvement in physical exercise. ANP levels in patients with coronary artery disease (CAD) are elevated, even in those treated with beta-blockers (Pierre Yves marine). In addition, individuals with CHF have elevated plasma levels of peptides N-terminal (NT) and pro-atrial peptide (pro-ANP) [10].

**Table 1:** Summary of the main studies regarding ANP and exercise.

*Author and year*	*Main findings*
**Moro et al, 2004 **[13]	Physical exercise induces the release of ANP, which stimulates lipolysis through a cGMP signaling pathway.
**Larsen et al, 2003 **[10]	Patients with chronic heart failure present an elevated plasma level of N-terminal peptide (NT) and pro-atrial peptide (pro-ANP) compared with healthy people.
**Marie et al, 2004 **[4]	Patients with coronary artery disease present high levels of ANP when treated with beta blockers.
**Melin et al, 2001 **[11]	During the process of exercise-induced dehydration there was a significant increase of ANP in relation to passively induced by heat.

According to Moro et al [11], exercise stimulates the production of ANP and hence lipolysis occurs via a cGMP signaling pathway. Another study found that subjects with CHF have high plasma levels of ANP also during the exercises. However, the high amount of circulating hormone is associated with a long maturity [10]. The process of exercise-induced dehydration is responsible for the increase of ANP induced by heat [12].

Acute infusion of pharmacological doses of ANP in hypertensive individuals decreases cardiac output and peripheral vascular resistance, suggesting an endogenous physiological role [3]. With respect to molecular effects, ANP stimulates the receptors of the plasma membrane of fat cells (NPR-A subtype), promoting an intrinsic activity of adenylate cyclase and increasing intracellular levels of cGMP, which activates a cGMP-dependent (PKG) phosphorylation of perilipin and HSL that stimulate lipolysis [13,14]. Moreover, within a physiological range, the ANP stimulates adipose tissue lipolysis, fat oxidation, and increases circulating levels of non esterified fatty acids (NEFA) [3].

Recent studies have shown that proANP1 and proANP31-30-67 exert a strong action on renal function through increased beta2-microglobulin, albumin and total protein excretion and inhibition of proximal tubular reabsorption [15]. Another important aspect is that when the intensity of exercise is increased, the blood flow decreases in the kidney in order to prevent dehydration, however, the water retention increases in the kidney. Moreover, after exercising a lot of protein comes from the circulation to the kidney, which comes from both the degradation of proteins damaged during exercise and negative nitrogen balance. Thus, the proANP proteirunic answer could be one mechanism to help the kidneys to excrete this amount of protein, protecting it from damage and dysfunction [1].

As mentioned above, the ANP is a hormone found in increasing concentrations in patients with CHF and is associated with functional impairment. There are some studies reporting increased plasma levels of ANP in response to dynamic exercise in these patients and lack of conditioning may be an important aggravating factor in the pathophysiology underlying the exercise [10]. During exercise, there is a high release of ANP in atrial secretory granules [10,16]. Nevertheless, patients with CAD have increased release of ANP when treatment includes beta-blockers. This secretion of vasodilatory and natriuretic constitutes a unique system for protecting the heart against stress [17].

The pro-ANP levels increased gradually in patients with CHF after doing exercises. The degree of increase in this hormone was obtained during exercise tests, and the result was associated with better survival in this population. These findings may represent a potential beneficial effect of increasing the capacity of ANP in CHF patients after completion of exercise [10]. In a previous study it was conducted a test exercise for 50 minutes at 60% VO_2_max and resting heat exposure. The results confirmed the significant increase in the amount of circulating ANP, confirming the action of this hormone in the renal system after completion of exercise [12].

Exercise is accepted as a strong stimulus for the release of alpha ANP. The dominant stimulus for its release is the atrial wall tension, a frequent phenomenon that occurs during exercise [1,17,18]. In addition, it was demonstrated that proANPs are deeply related to other hormonal systems involved in physical exercise [1,10,12,19]. A study measured the urinary levels of proANP1-30-67 and proANP31 before and after strenuous exercise. They showed that urinary levels of proANP1-30 were not different between before and after exercise, while pro ANP31-urinary-67 showed a significant increase [1].

ANP reduces blood pressure by decreasing the sensitivity of vascular smooth muscle to the action of vasoconstrictors and regulate fluid balance. This secretion of potent vasodilating and natriuretic agents constitutes an original therapeutic mechanism for the protection of heart disease and cardiac stress though high for both peptides (ANP and BNP) in patients with heart failure and more limited in normal subjects [4]. On the other hand, according to previous studies, the elevation of ANP at rest was associated with a higher risk of mortality. The increase during the exercise test was associated with better survival in the study population, indicating a physiological benefit and an efficient mechanism to increase the ability of ANP during exercise in patients with CHF. Therefore, although several studies show an association between elevated levels of ANP and disease progression in patients with CHF, it was suggested that this neuro-hormone is a prognostic marker, which may also mediate the effects of the benefit these patients [10].

Some findings were observed in studies of patients with cardiovascular disease, especially congestive heart failure (CHF), where it was selected 25 patients with coronary artery disease, they were submitted to exercise stress test on a treadmill, and after blood collection and analysis, the presence of pre ANP hormone, increased in concentration by increasing the survival of these patients [10]. In patients with chronic CAD, the average concentration of ANP was two times higher than in normal subjects after exercises and use of beta-blockers [17]. Although it is necessary further studies regarding the cardiac function and its relationship with the ANP, the ANP function presents itself as an indicator of the degree of functionality of cardiac function in patients with cardiac disease in particular. The presence of this circulating hormone may be a preventive tool in the development of chronic diseases related to the cardiovascular system [10].

The ANP also inhibits the function of various other hormones such as aldosterone, angiotensin II, endothelin, renin and vasopressin. In the kidneys, it inhibits the absorption of sodium in the collecting ducts of nephrons, it inhibits the action of aldosterone and counteracts the renin-angiotensin-aldosterone system. Consequently, it increases excretion of sodium. As a consequence, water follows sodium because of osmosis. The alfa ANP with other proANP fragments (proANPs) exerts a strong influence on renal function. In fact, it increases glomerular filtration rate, inhibiting the NAQ transport and increase the volume of urine and excretion of protein [1,3,15]. In a study of 28 athletes cyclists, urine samples were collected before and after the test and the result was positive for a high concentration of fragments of the pro-hormone (pro ANP1 pro-30 and 31-67) with other proteins, which has a strong influence on the renal system, increasing the rate of glomerular filtration. The reason for this is that these particular pre-hormones have effects on specific binding region of the medullary duct, and increased physical effort practiced by cyclists increased renal filtration increases, occurring an abrupt overload, but the high concentration of ANPs facilitates the filtration rate of many proteins because its vasodilator effect and decreasing renal overload [18].

In addition to catecholamines, insulin and ANP are essential physiological regulators of lipolysis of human adipocytes [3,20]. It was demonstrated in a study that the physiological release of ANP during the acute physical exercise contributes to the stimulation of lipid mobilization and supply of NEFA to the working muscles [21]. Some studies have shown higher levels of ANP at rest in trained animals and humans [22] and others did not report any effect of training [12].

Patients with CHF have high level of N-terminal peptide (NT) and pro-atrial peptide (pro-ANP) in plasma compared with healthy people, and this neuro-hormone concentration is even higher during exercise, which can be associated with better survival in this population, suggesting a great benefit and increased potential to increase the amount of ANP [10]. Furthermore, patients with CAD have increased concentrations of ANP after conducting exercises when they are treated with beta-blockers. This secretion of potent vasodilating and natriuretic agents constitutes an original therapeutic mechanism for protection of the diseased heart against stress. During exercise, the release of secretory granules allows ANPs by an immediate increase in blood levels of this neurohormone. These granules are located mainly in atrial myocytes, but also located in ventricular myocytes in patients with congestive heart failure [17]. In addition, during the processes of dehydration, plasma ANP increased significantly during exercise-induced dehydration, whereas it did not change during passive heat-induced dehydration [12]. Nonetheless, more studies are necessary to better understand the exact role of exercise in proANP fragment. In particular, proANP1, proANP31-30-67 and urinary tract can contribute to a long-term local involvement of ANP in the regulation of kidney function [3].

## Concluding Remarks

Our review provides new evidence based on recent studies related to the beneficial effects of exercise in patients with cardiovascular disease, focusing on the ANP. This minireview does not allow us to accurately identify all the mechanisms involved in changes in plasma ANP levels induced by exercise. The current literature shows a direct correlation between the release of ANP induced by exercise and reduced cardiovascular mortality. Further studies investigating non-pharmacological therapies such as exercise, are always welcome in the literature.

## Competing interests

The authors declare that they have no competing interests.

## Authors' contributions

All authors participated in the acquisition of data and revision of the manuscript. All authors conceived of the study, determined the design, performed the statistical analysis, interpreted the data and drafted the manuscript. All authors read and gave final approval for the version submitted for publication.
